# Recognition of Membrane Sterols by Polyene Antifungals Amphotericin B and Natamycin, A ^13^C MAS NMR Study

**DOI:** 10.3389/fcell.2016.00057

**Published:** 2016-06-17

**Authors:** Filip Ciesielski, David C. Griffin, Jessica Loraine, Michael Rittig, Joss Delves-Broughton, Boyan B. Bonev

**Affiliations:** ^1^School of Life Sciences, Queen's Medical Centre, University of NottinghamNottingham, UK; ^2^Health and Nutrition, DuPont UK LtdBeaminster, UK

**Keywords:** antimicrobials, antifungals, membranes, cholesterol, ergosterol, ^13^C solid state MAS NMR, receptor recognition

## Abstract

The molecular action of polyene macrolides with antifungal activity, amphotericin B and natamycin, involves recognition of sterols in membranes. Physicochemical and functional studies have contributed details to understanding the interactions between amphotericin B and ergosterol and, to a lesser extent, with cholesterol. Fewer molecular details are available on interactions between natamycin with sterols. We use solid state ^13^C MAS NMR to characterize the impact of amphotericin B and natamycin on mixed lipid membranes of DOPC/cholesterol or DOPC/ergosterol. In cholesterol-containing membranes, amphotericin B addition resulted in marked increase in both DOPC and cholesterol ^13^C MAS NMR linewidth, reflecting membrane insertion and cooperative perturbation of the bilayer. By contrast, natamycin affects little either DOPC or cholesterol linewidth but attenuates cholesterol resonance intensity preferentially for sterol core with lesser impact on the chain. Ergosterol resonances, attenuated by amphotericin B, reveal specific interactions in the sterol core and chain base. Natamycin addition selectively augmented ergosterol resonances from sterol core ring one and, at the same time, from the end of the chain. This puts forward an interaction model similar to the head-to-tail model for amphotericin B/ergosterol pairing but with docking on opposite sterol faces. Low toxicity of natamycin is attributed to selective, non-cooperative sterol engagement compared to cooperative membrane perturbation by amphotericin B.

## Introduction

Amphotericin B and natamycin belong to the class of macrolide polyene antibiotics, which are used to prevent and treating opportunistic fungal infections, as well as in the control of food spoilage. Both antimycotic compounds are membrane-active and have been shown to interact with ergosterol (Gabrielska et al., 2006; Matsuoka et al., 2006). While amphotericin B uses ergosterol as a receptor for membrane disruption, natamycin does not permeabilise membranes directly but undermines the function of ergosterol-dependent membrane transport.

Amphotericin B is produced by *Streptomyces nodosus* and kills fungal cells by forming membrane pore complexes with ergosterol. The molecule is approximately 23 Å long, which is slightly less than the typical membrane thickness and its activity is sensitive to lipid chain length (Matsuoka et al., 2006). Solid state NMR has been used to show that amphotericin B can form a single ring spanning the lipid bilayer when the drug is delivered from one side of a membrane (Gabrielska et al., 2006) or a double length ring when it is present on both sides of a membrane (Matsuoka et al., 2005). Treatment with amphotericin B is restricted to a minimum due to its severe side effects, such as nephrotoxicity. However, due to lack of alternatives, for the past 50 years it remains one of the most common antimycotics. There is a great interest in understanding its mechanism of action and factors which affect the specificity toward fungal cells as this knowledge could allow designing more efficient and specific drugs. Studies with red blood cells showed that amphotericin B can target cholesterol-containing membranes (Kotler-Brajtburg et al., 1974), which is thought to be the primary cause of its toxicity. However, experiments also revealed that amphotericin B has greater affinity to ergosterol than cholesterol (Readio and Bittman, 1982), partially explaining the specificity of the drug. However, because the channel formation involves eight amphotericin B molecules and eight sterols, Baginski group (Baginski et al., 2002) have speculated that small differences in sterol affinity cumulate and become significant enough to trigger specificity. Their molecular simulations showed that channels formed with ergosterol are bigger than those formed with cholesterol, greatly affecting the ion permeability.

A great deal of attention has been also focused on the two sterols. Both are known to induce liquid ordered phases on lipid bilayers where trans-gauche isomerizations vanish (Endress et al., 2002; Mouritsen and Zuckermann, 2004; Rog et al., 2009), however, the degree of ordering depends on the lipid type. For example ergosterol has a stronger ordering effect on the saturated lipid chains than cholesterol, but the opposite is true for unsaturated phospholipid bilayers (Urbina et al., 1998). Furthermore, quantum calculations showed significant differences in electric potentials distributions between ergosterol and cholesterol. Highly negative regions were predicted in the ergosterol, which could account for its higher affinity toward the polar macrolide structures within a hydrophobic environment of a lipid bilayer (Baginski et al., 1997a). In addition, molecular dynamics simulations have showed that ergosterol tail chain is stiffer and more elongated than that of cholesterol, making the former more stable and providing stronger van der Waals contacts with the drugs.

Other studies, on the other hand, suggest that cholesterol is not required for the amphotericin B-driven formation of pores. Cotero et al. (1998) found that ionic current can be detected on amphotericin B addition without the presence of sterols in a membrane. They hypothesized that the role of a sterol in the channel formation is via the effect it has on the membrane itself, instead of directly affecting the channel formation. Furthermore, molecular simulations also backed this view, showing that cholesterol does not even interact with amphotericin B at all in any specific way (Baginski et al., 1997b).

Natamycin is produced by *Streptomyces natalensis* and is used on a commercial scale in the food industry as preservative E235 and also as an additive to topical antifungal creams. In contrast to Amphoteracin B, natamycin has been less well-characterized. Previous views that natamycin shares mode of action with amphotericin B, i.e., pore formation and dissipation of ions and cell death have been overturned by observations that natamycin does not form membrane pores but interacts specifically with ergosterol excluding it from membrane functionality (te Welscher et al., 2008) and preventing vacuolar trafficking (te Welscher et al., 2010) and endocytosis (Van Leeuwen et al., 2009). Recent studies develop a clearer mechanistic picture, in which natamycin/ergosterol interactions downregulate ergosterol-dependent membrane transport proteins (te Welscher et al., 2012).

While interactions of amphotericin B and, to a lesser extent of natamycin with ergosterol have already been studied in detail in the past (Fournier et al., 1998, 2008; Gagos et al., 2005; Matsumori et al., 2006; Kasai et al., 2008; Gruszecki et al., 2009; Foglia et al., 2012; Gagos and Arczewska, 2012; Umegawa et al., 2012), less is known about their interactions with cholesterol. In this study we use solid state magic angel spinning (MAS) NMR to investigate the effects of amphotericin B and natamycin on mixed lipid membranes containing DOPC and cholesterol or ergosterol. Natural abundance ^13^C is used in MAS NMR to observe the effects of each antifungal on lipid and sterol dynamics and the differential effect of amphotericin B and atamycin on membranes containing cholesterol or ergosterol.

## Materials and methods

### Sample preparation

Lipids were purchased and used without further purification, 1,2-di-oleoylphosphatidylcholine (DOPC) from Avanti Polar Lipids; cholesterol from BDH chemicals Ltd Poole England; and, 98% Ergosterol (nitrogen fixed) from Acros Organics. Amphotericin B was purchased from MP Biomedicals and used without further purification. Natamax was kindly donated by DuPont and lactose was removed *via* multiple washes in distilled water to yield pure natamycin.

DOPC was mixed with either cholesterol or ergosterol in 7:3 ratios in chloroform/methanol and re-suspended in a working buffer 10 mM HEPES pH 7.0, 10 mM NaCl, 1 mM EDTA (the same buffer was used for all samples). Natamycin was dissolved in methanol and added to the DOPC/sterol lipid film (final ratio of DOPC/sterol/drug was 7:3:1.5), and after solvent removal also re-suspended in the working buffer. Amphotericin B was dissolved in di-methylformamide (DMF) and added to DOPC/Cholesterol lipid film (final ratio 7:3:1.5) and DMF was removed by repeated 7 × dilution with distilled water and centrifugation at 20,000 rpm at 20°C (Beckman coulter Avanti J25) for 30 min. Residual DMF was removed under high vacuum and the sample was re-suspended in the working buffer. All samples were then freeze-thawed a minimum of 5 times each, pelleted at 16,060 g for 15 min (Biofuge pico, Heraeus) and the pellets were loaded in 4 mm MAS NMR rotors.

### Solid state NMR spectroscopy

All solid-state NMR experiments were performed on a Varian 400 MHz (VNMRS) spectrometer using a 4 mm T3 MAS NMR probe (Varian, Palo Alto CA, USA). Cross-polarization, CP, (Hartmann and Hahn, 1962) with proton ramp (Metz et al., 1994) was used to prepare observable ^13^C magnetization. A 96 kHz proton excitation pulse was followed by 3.5 ms of 45 kHz Hartmann-Hahn contact. MAS NMR spectra were acquired using 60 kHz SPINAL decoupling (Fung et al., 2000) to suppress heteronuclear ^1^H−^13^C dipolar interactions or using 96 kHz FSLG decoupling (Bielecki et al., 1989). Acquisitions were repeated each 3.5 s with 150 ms acquisition times under SPINAL and 75 ms duration under FSLG. To obtain heteronuclear-decoupled high-resolution spectra, 1024 transients were averaged, while 8192 transients were added when homonuclear decoupling was used. Sample temperature was regulated using balanced heated/vortex tube-cooled gas flow (Ciesielski et al., 2009) and MAS/RF-induced temperature elevation of 4°C was taken into consideration by indirect reference to lipid main transition suppression in DMPC and DPPC liposomes. Sample rotation was set at 5 kHz, which is sufficient to produce well-averaged high-resolution spectra in mobile hydrated lipid systems. Spectral prediction and processing was done using ADC Labs (Toronto, Canada).

## Results

### Molecular interactions of amphotericin B and natamycin with membrane sterols

**Figure 1:**
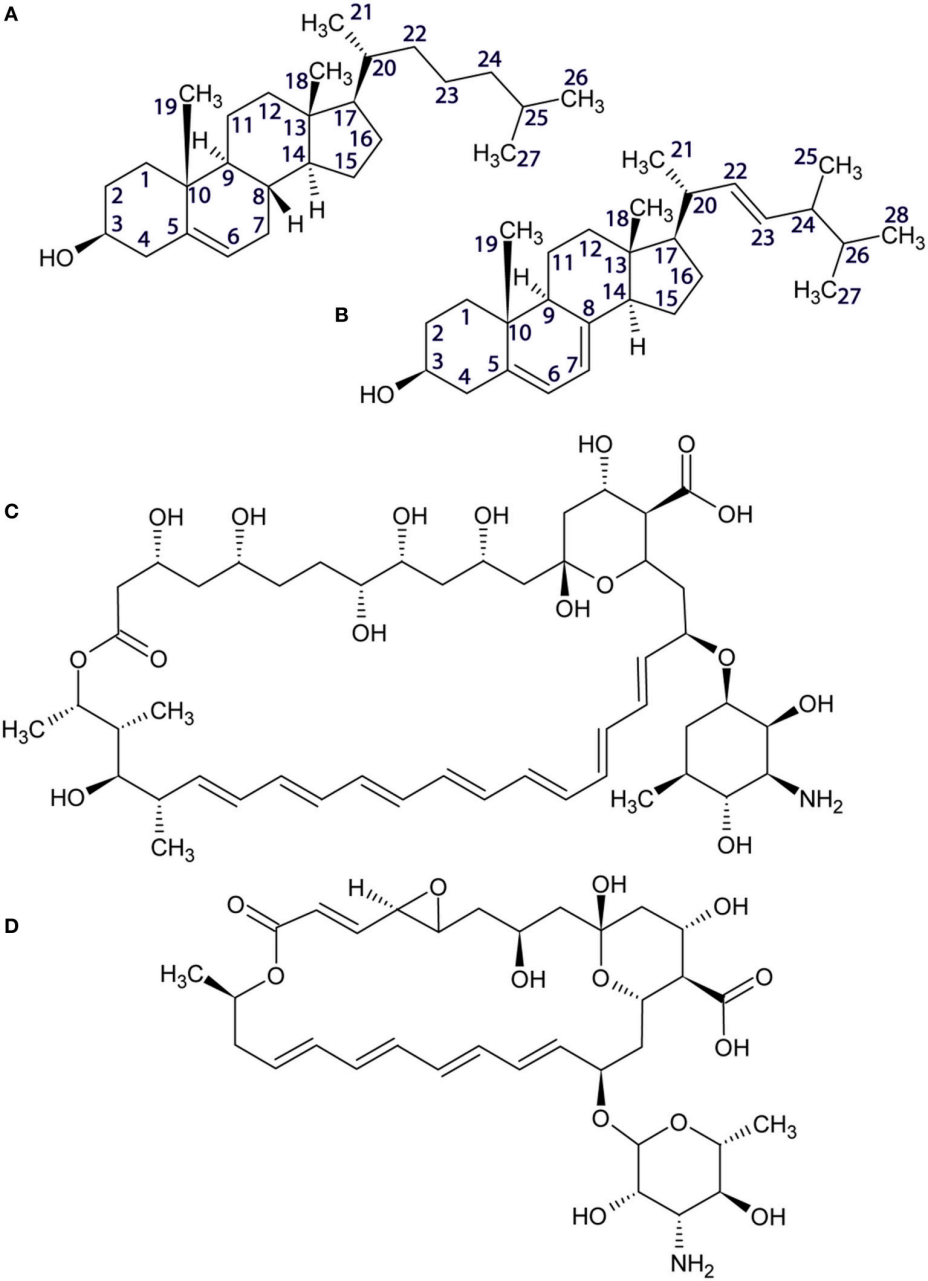
**Structures of cholesterol (A), ergosterol (B), amphotericin B (C), and natamycin (D)**.

**Figure 2:**
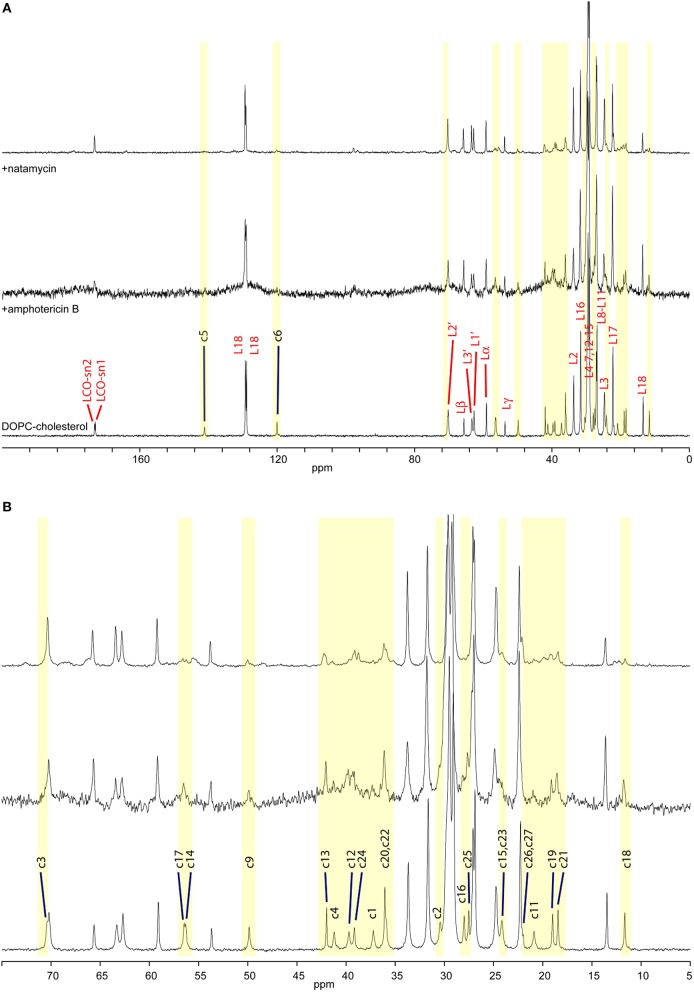
**High-resolution ^**13**^C MAS NMR spectra of hydrated DOPC/cholesterol membranes: (A) full spectrum showing lipid resonances and some cholesterol resonances; membrane alone (bottom); with amphotericin B (middle); and with natamycin (top); (B) aliphatic/ring region expanded, showing cholesterol assignment**.

**Figure 3:**
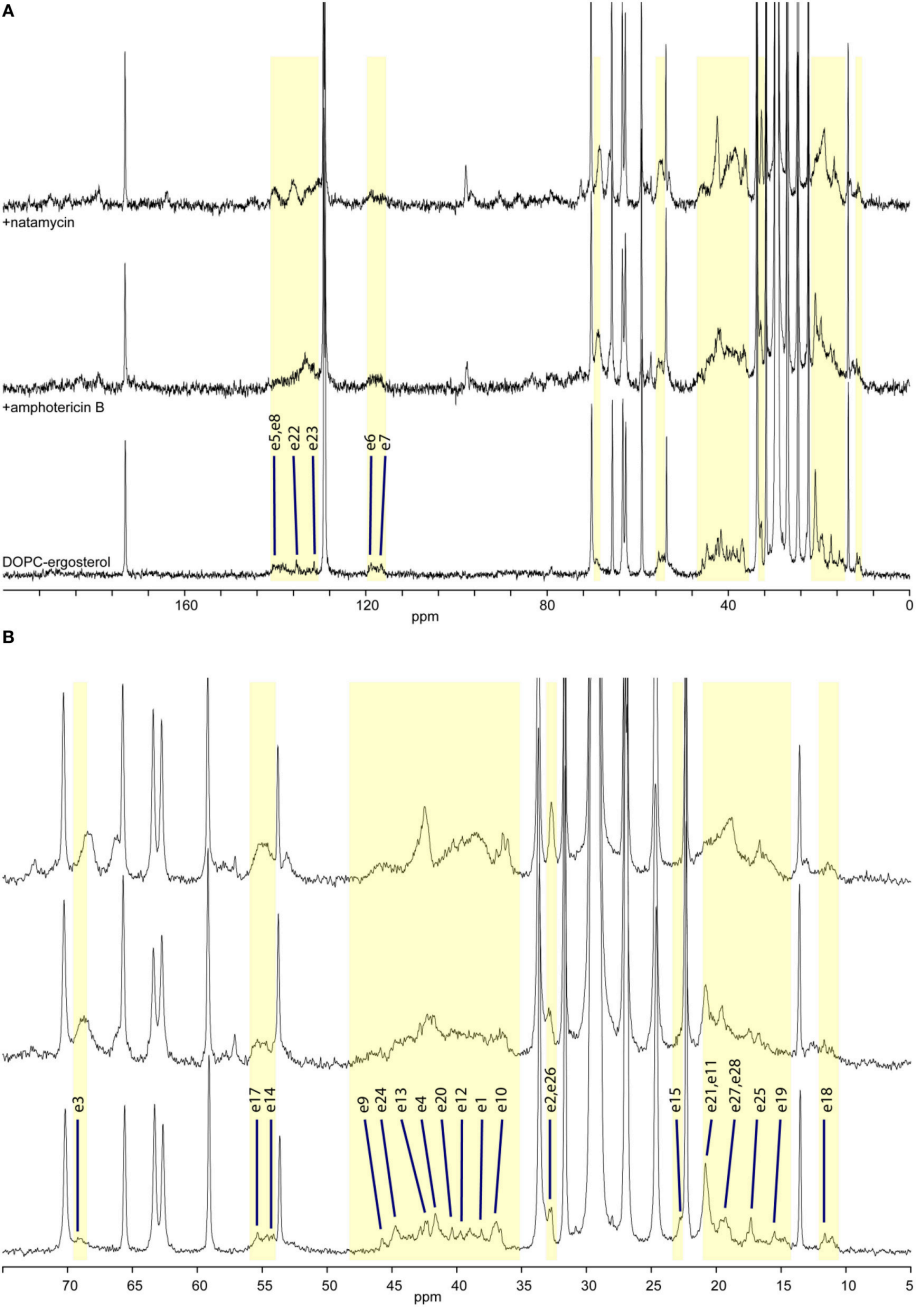
**High-resolution ^**13**^C MAS NMR spectra of hydrated DOPC/ergosterol membranes: (A) full spectrum showing lipid resonances and some ergosterol resonances; membrane alone (bottom); with amphotericin B (middle); and with natamycin (top); (B) aliphatic/ring region expanded, showing ergosterol assignment**.

High-resolution ^13^C MAS NMR spectra, acquired under SPINAL decoupling from DOPC liposomes containing 30% sterol at 20°C yielded well-resolved resonances. Cholesterol and ergosterol structures are shown in Figure 1 alongside atom numbering used in resonance assignments (Zorin et al., 2010). Pure DOPC membranes were characterized for reference. Samples made with cholesterol produced well-resolved lipid and sterol resonances (Figure 2), which were assigned following (Forbes et al., 1988; Zorin et al., 2010). DOPC/ergosterol samples produced well-resolved lipid peaks but broad and low-intensity ergosterol resonances in most cases (Figure 3), due to intermediate mobility of this sterol in DOPC membranes, which interferes with cross-polarization and homonuclear dipolar decoupling. Resolved ergosterol peaks were assigned according to $Soubias et al. (Soubias and Gawrisch, 2005) and used to monitor the membrane response to antifungals. The same set of samples was studied using ^13^C MAS NMR spectroscopy with FSLG decoupling during signal acquisition, which removes proton degeneracy and provides J-resolved ^13^C spectra for sufficiently mobile molecular segments. Well-resolved couplings were measured for DOPC resonances only, due to the poor resolution of the sterol multiplets.

Molecular interactions in membranes can be followed using ^13^C MAS NMR by following changes in segmental dynamics. Such changes impact longitudinal relaxation, which leads to loss of intensity in resonances from directly interacting groups (Sanghera et al., 2011) or intensity enhancement under CP-MAS where fast molecular mobility interfering with CP or decoupling is moderated in a complex and NMR signal is enhanced. Figure 2 shows ^13^C MAS NMR spectra from DOPC/cholesterol membranes alone (bottom) and in the presence of amphotericin B (middle) or natamycin (top). Amphotericin has a profound effect on membrane dynamics and all lipid and cholesterol resonances appear broadened, indicating that amphotericin B does insert in the membrane and that insertion has a disordering effect leading to increased librational freedom for both DOPC and cholesterol. The linewidth is likely related to motional interference with the proton decoupling, as the lipid CO resonances, which have no directly bonded protons appear less affects. Despite this line broadening, the relative intensity of cholesterol resonances compared to DOPC does not appear significantly altered, which indicates no preferential interaction of amphotericin B with cholesterol over DOPC.

While fast lipid dynamics is dominated by cooperative axial rotation, changes in segmental mobility in response to antifungals can still be observed by ^13^C solid state NMR and segmental dynamics in sterols is even more informative. In the presence of amphotericin B cholesterol core residues c1–6, c9, c11, c14, c15–17 are significantly attenuated, indicating reduced core mobility (Figure 2 middle). Curiously, c12 is attenuated but still observed and methyls c18, c19, and c21, which line the same region of the cholesterol molecule do not appear affected. Amphotericin has little effect on chain residues c20–c25, as well. However, resonance intensity from chain terminal methyl groups c26 and c27 are enhanced, which points to participation of the chain end in the interaction between amphotericin and cholesterol. Chain terminal coupling has been reported between ergosterol terminal methyl groups e27, e28 and amphotericin B in their membrane complex (Umegawa et al., 2012). Together, the data put forward an interaction model, in which the cholesterol ring core plays the leading role in interactions with amphotericin B, chiefly on the c4, c6, c15 ridge and with engagement of the chain end c26, c27.

The impact of natamycin on the spectrum from DOPC/cholesterol membrane is quite different (Figure 2 top). Overall, resonances have retained their width offering good spectral resolution. The impact of natamycin on cholesterol mobility indicates the formation of a molecular complex, which bears resemblance to that between amphotericin B and cholesterol showing ring core and chain-terminal interactions with natamycin. However, distinct differences in segmental dynamics reveal mobility restrictions on all ring methyl groups, as well as on ring A (c1–5, c10) and the base of the chain. This points to natamycin engagement of the cholesterol molecule on the opposite side to that with amphotericin B and involving cholesterol ring 1 (c1–c5) and the chain end (c25–c27), however, also with contact on methyls c18, c18 and not directly with the other rings.

Ergosterol-containing membranes undergo motions near the NMR timescale, which leads to some interference with proton decoupling and cross-polarization and erodes some ergosterol and antibiotic resonances from the ^13^C MAS NMR spectra (Figure 3, bottom). Resolved resonances inform on the impact of antibiotic presence on segmental dynamics through changes in resonance intensity (dependent on the longitudinal relaxation time T_1_) by comparison to the repeat delay.

The presence of amphotericin B in the DOPC/ergosterol membrane is associated with attenuation of ergosterol resonances, which is consistent with altered molecular dynamics during complex formation between ergosterol and amphotericin B. This attenuation is most noticeable and specific for resonances e1, e9, e12, and e19, which complement the interfacial ridge of rings A, B, and C in ergosterol along with methyl e19. The opposite side of the molecule, e15, is also attenualted, which appears to link to mobility restrictions in the chain at e20, e22, e24, and e25 (Figure 3, middle). The terminal methyls e27 and e28 are not attenuated but are shifted. This points to an interaction picture, which involves the e1-e9-e12 edge with e19 and the beginning of the chain e20, e22, e24 along with ring D at e15 (Figure 1).

Addition of natamycin to ergosterol/DOPC membranes yields a better resolved spectrum, in which a number of natamycin residues are also observed (Figure 3, top). The attenuation of ergosterol signals in this spectrum is far more widespread involving the same ergosterol ridge e1, e10, e9, e11, as well methyl e19 but also resonances e3 and e4 on ring A, e7 on the opposite side of ring B, e13 and e15 on ring D. Interestingly, akin to amphotericin, natamycin does not attenuate methyl e18. The ergosterol chain is also more profoundly affected with resonances e23, e24, and e25 attenuated, as well as terminal methyls e27 and e28. Curiously, resonance e26 is significantly enhanced, showing that the end of the ergosterol chain undergoes non-cooperative motions near the intermediate timescale that are affected profoundly on natamycin binding. In contrast to amphotericin B, antamycin appears to engage methyl e21 at the beginning of the chain (Figure 1). The impact of natamycin on mobility at e5 and e8 is difficult to assess due to overlap with natamycin polyene chain signal. Similarly, e14 and e17 resonances are no longer resolved and are obscured by the epoxyde carbons from natamycin (Figure 3, top).

The changes in ergosterol segmental mobility in the presence of natamycin along with the persistent spectral resolution indicate a more specific antibiotic/sterol molecular interaction, somewhat resembling that between amphotericin and ergosterol (Umegawa et al., 2012). Such molecular complexes appear to have comparatively little effect on lipid and sterol cooperative dynamics. By contrast, while the observed spectroscopic changes in amphotericin B/ergosterol spectra support the molecular model, proposed in (Umegawa et al., 2012), they also suggest that the effect is far more cooperative affecting the whole membrane, while the natamycin/ergosterol complexes appear to interact with each other and with the membrane bulk much less and, subsequently, perturb membrane lipid to much lesser extent. The significance of this observation is that while amphotericin B at a mechanistic level may affect the functionality of ergosterol-dependent proteins through significant modulation of membrane properties (te Welscher et al., 2008), natamycin appears to have a very specific effect on ergosterol, which is not transmitted to the rest of the membrane.

### Effects of amphotericin B and natamycin on membrane phospholipid

Besides specific interactions with sterols, followed directly from changes in ^13^C MAS NMR spectra from cholesterol and ergosterol, the addition of polyene antibiotics also affects membrane phospholipid. Within hydrated membranes, phospholipids undergo fast cooperative axial motions with GHz rates and the full width at half height (FWHH) of lipid resonances is affected by the cooperative mobility, fast axial rotation, and slow angular librations, as well as by additional, individual segmental motions. The presence of additional membrane constituents can affect any or many of these motions and the effects are seen in the high-resolution ^13^C MAS NMR spectra from the membrane lipid (Ciesielski et al., 2009; Zorin et al., 2010). In addition, the isotropic chemical shifts of each carbon environment can be affected directly by the proximity of membrane active molecules or indirectly by changes in molecular order parameters and packing (Bonev et al., 2001).

Lipid resonance full width at half height (FWHH) is affected primarily by lateral molecular librations or rocking and increases in membranes with lower bending modulus, where membrane undulation is facilitated by lose chain packing (Ciesielski et al., 2009). By contrast, the presence of cholesterol stiffens the membrane and improves packing in the chain region without impairing axial lipid rotation, which leads to significant reduction of linewidth and improved spectral resolution (Zorin et al., 2010). Resolution can also be improved under proton decoupling and mobility that interferes with decoupling can increase linewidth. Thus, the FWHH is related to the transverse relaxation rate and a good indicator of changes in librational mobility of lipids in response to membrane interactions.

**Figure 4:**
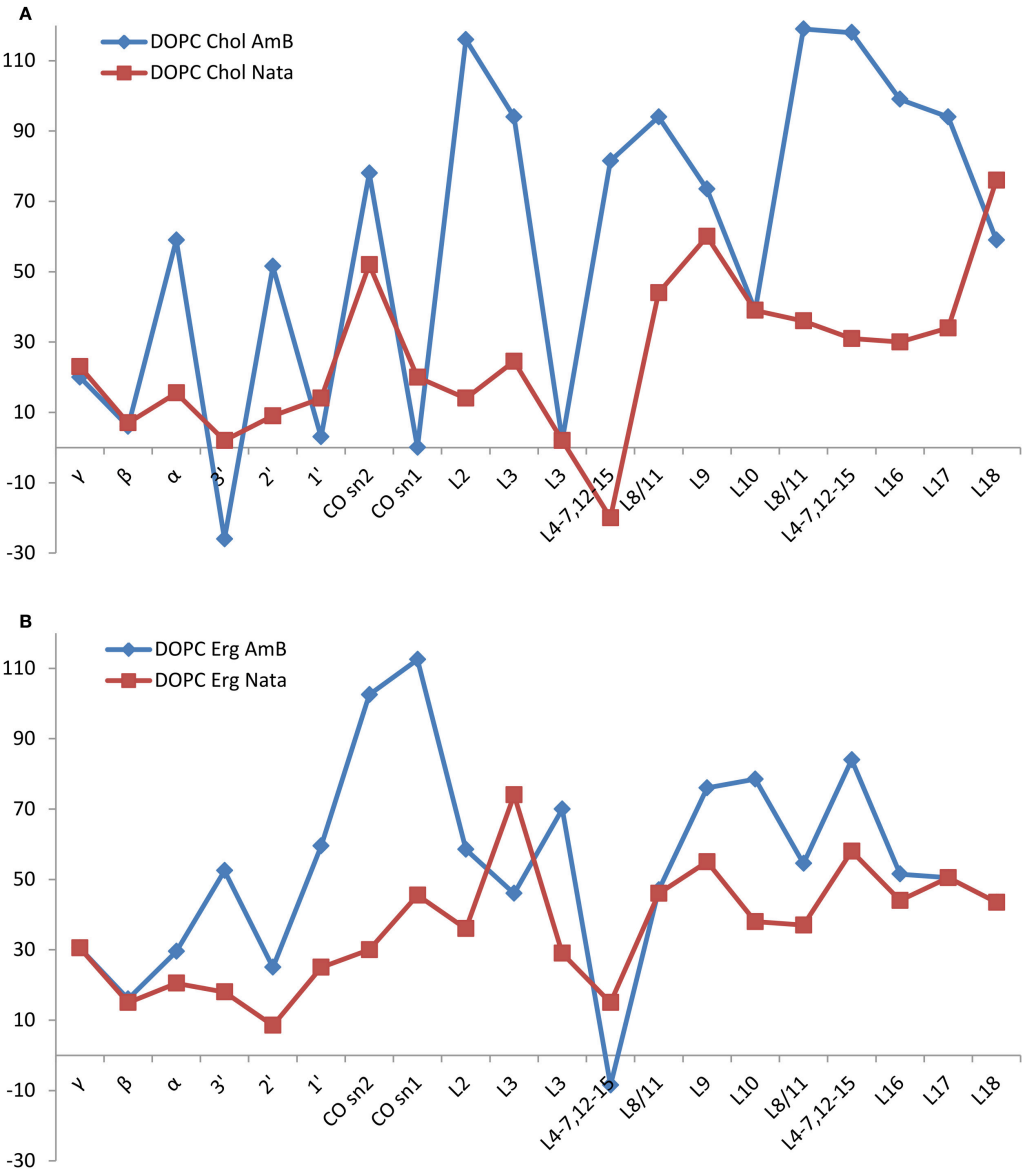
**Changes in DOPC resonance linewidth on addition of amphotericin B (red squares) or natamycin (blue diamonds) with respect to DOPC chemical shifts in DOPC/cholesterol (A) or DOPC/ergosterol (B) membranes, respectively**.

The changes in DOPC ^13^C linewidth in mixed membranes with cholesterol or ergosterol before and after addition of amphotericin B or natamycin are shown in Figure 4 alongside the carbon number. Addition of amphotericin B to cholesterol containing DOPC mixed membranes leads to marked increase in linewidth, particularly in chain region, indicative of incorporation of the amphotericin B into the hydrophobic core of the membrane. The effect is consistently observed throughout chain methylenes, reflective of the cooperativity of chain motions. Broadening of carbonyl and to a lesser extent backbone and headgroup resonances arises from some degree of cooperative mobility of the whole DOPC molecule. Similar broadening of chain resonances is observed in the presence of natamycin but to a lesser extent, consistent with the comparatively less perturbing insertion of natamycin into the membrane.

Both amphotericin B and natamycin broaden DOPC resonances in ergosterol/DOPC mixed membranes. Reflective of reduced axial rotation and enhanced librational freedom, the effect is more spread out over the entire lipid molecule with a notable significant broadening of the carbonyl resonances by amphotericin B. Overall, backbone and headgroup resonances remain comparatively less perturbed, which confirms insertion of both antifungals into the membrane interior.

**Figure 5:**
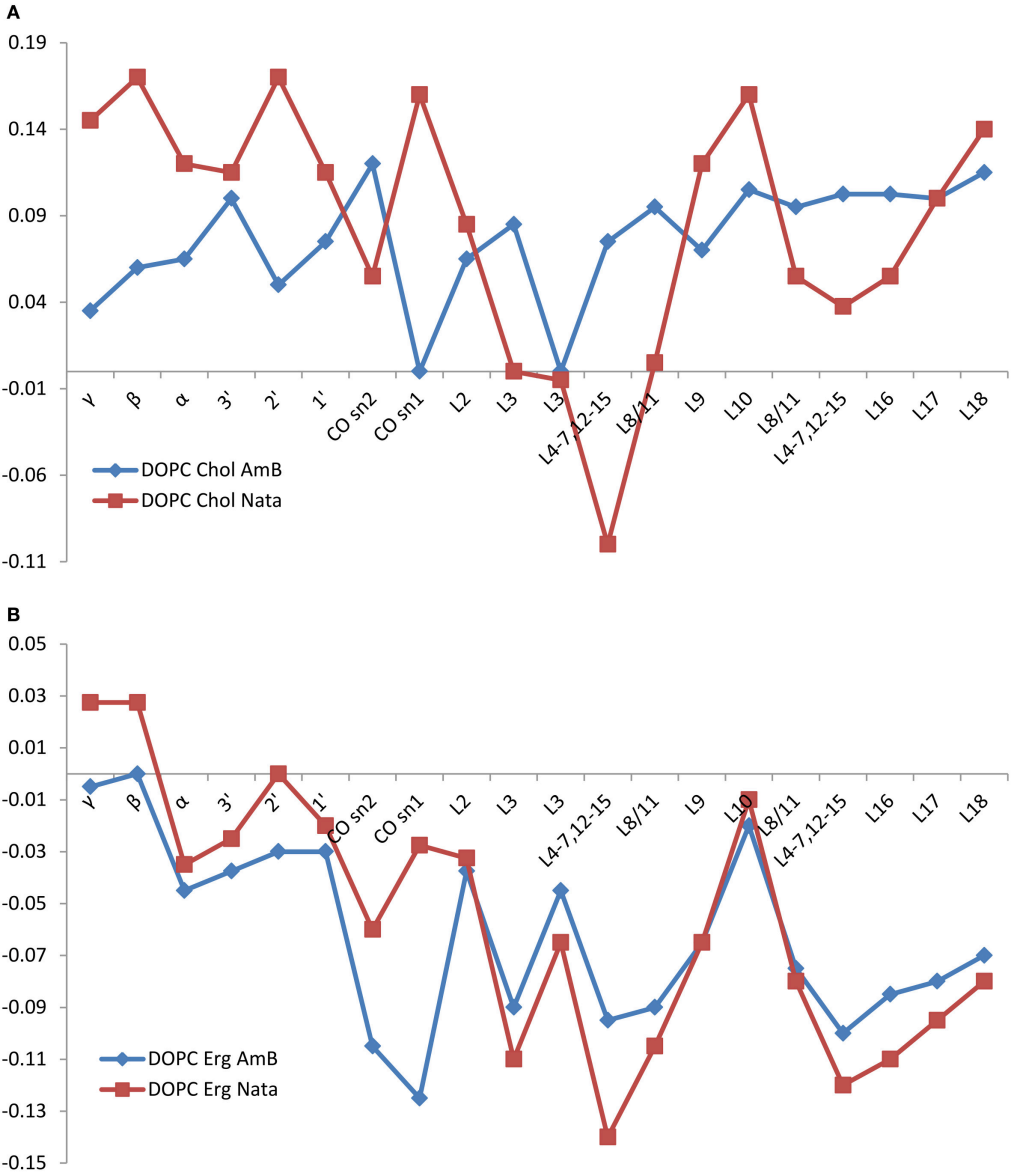
**Changes in DOPC resonance isotropic ^**13**^C chemical shift on addition of amphotericin B (red squares) or natamycin (blue diamonds) with respect to DOPC chemical shifts in DOPC/cholesterol (A) or DOPC/ergosterol (B) membranes, respectively**.

The presence of amphotericin B or natamycin has a minor impact on DOPC ^13^C isotropic chemical shifts in either cholesterol or ergosterol-containing membranes (Figure 5). In DOPC/cholesterol membranes natamycin addition leads to more pronounced changes in chemical shifts (Figure 5A). This is likely the result of the described earlier specific interactions with cholesterol, which lead to altered order in the DOPC component of the mixed membrane and more pronounced changes in the headgroup region. The effects on DOPC/ergosterol membranes are very similar and support the picture of collective molecular organization within the membrane, discussed earlier.

**Figure 6:**
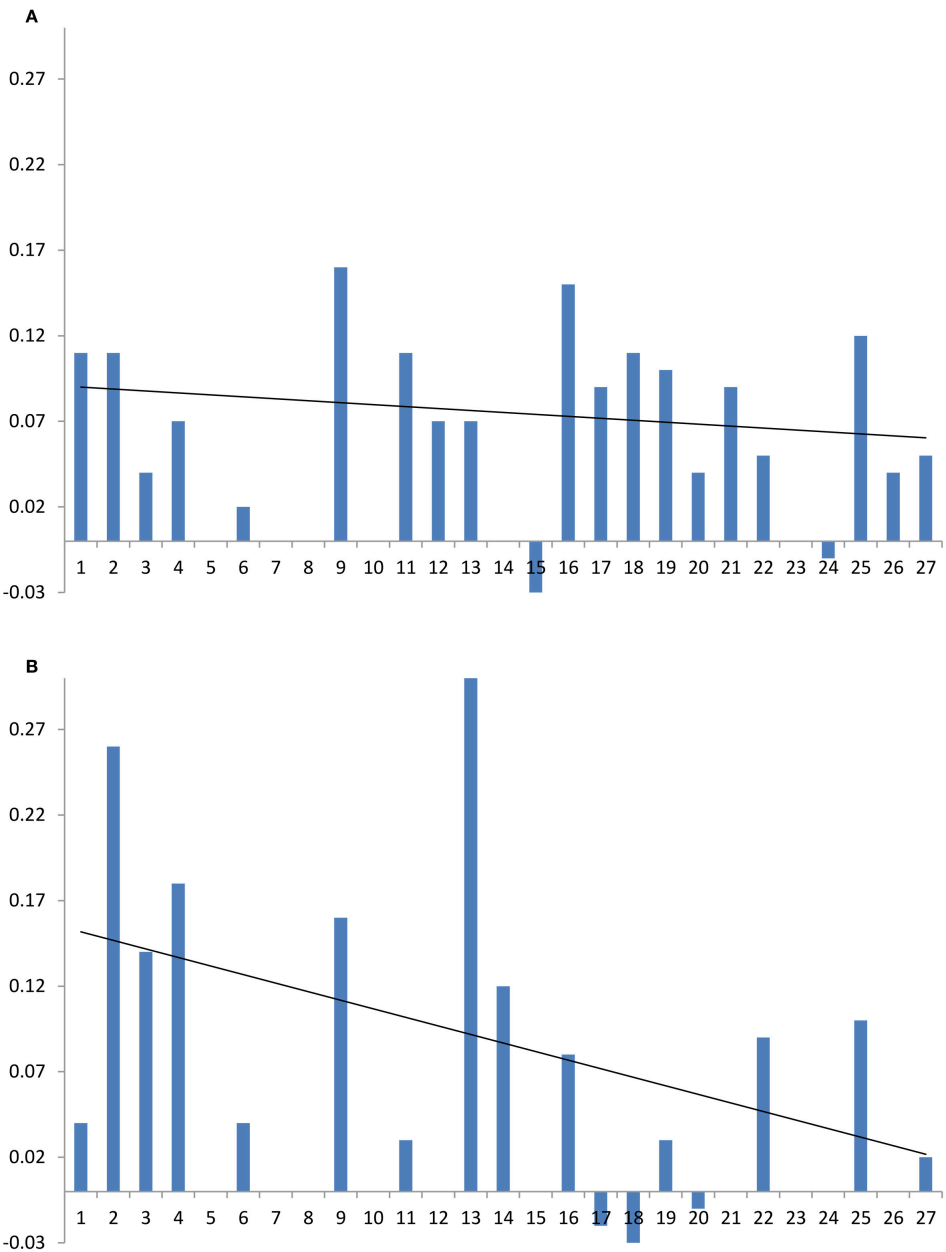
**Changes in cholesterol ^**13**^C chemical shift due in the presence of antifungal compounds: (A) amphotericin B, (B) natamycin**. Carbon numbers, shown on the abscissa, correspond to those in Figures 1, 7 and roughly follow end-to end direction on the sterol molecule. Trendlines guide the eye to approximate end-to-end chemical shift changes along each sterol molecule.

Good spectral resolution in DOPC/cholesterol membranes allows monitoring the changes in the ^13^C isotropic chemical shifts of cholesterol, as well. The presence of amphotericin B in the DOPC/cholesterol membrane has a small and comparatively uniform impact on ^13^C chemical shifts of cholesterol (Figure 6A), which reflects the observed comparatively small impact on longitudinal relaxation and linewidth (transverse relaxation). This offers further support to a cooperative and non-specific effect of amphotericin on DOPC/cholesterol membranes. The addition of natamycin to DOPC/cholesterol membranes has more location-specific impact on ^13^C chemical shifts, which correlates well with resonances proximal to the observed resonance attenuation at the sterol core, core methyls and the end of the chain (Figure 6B).

## Discussion

**Figure 7:**
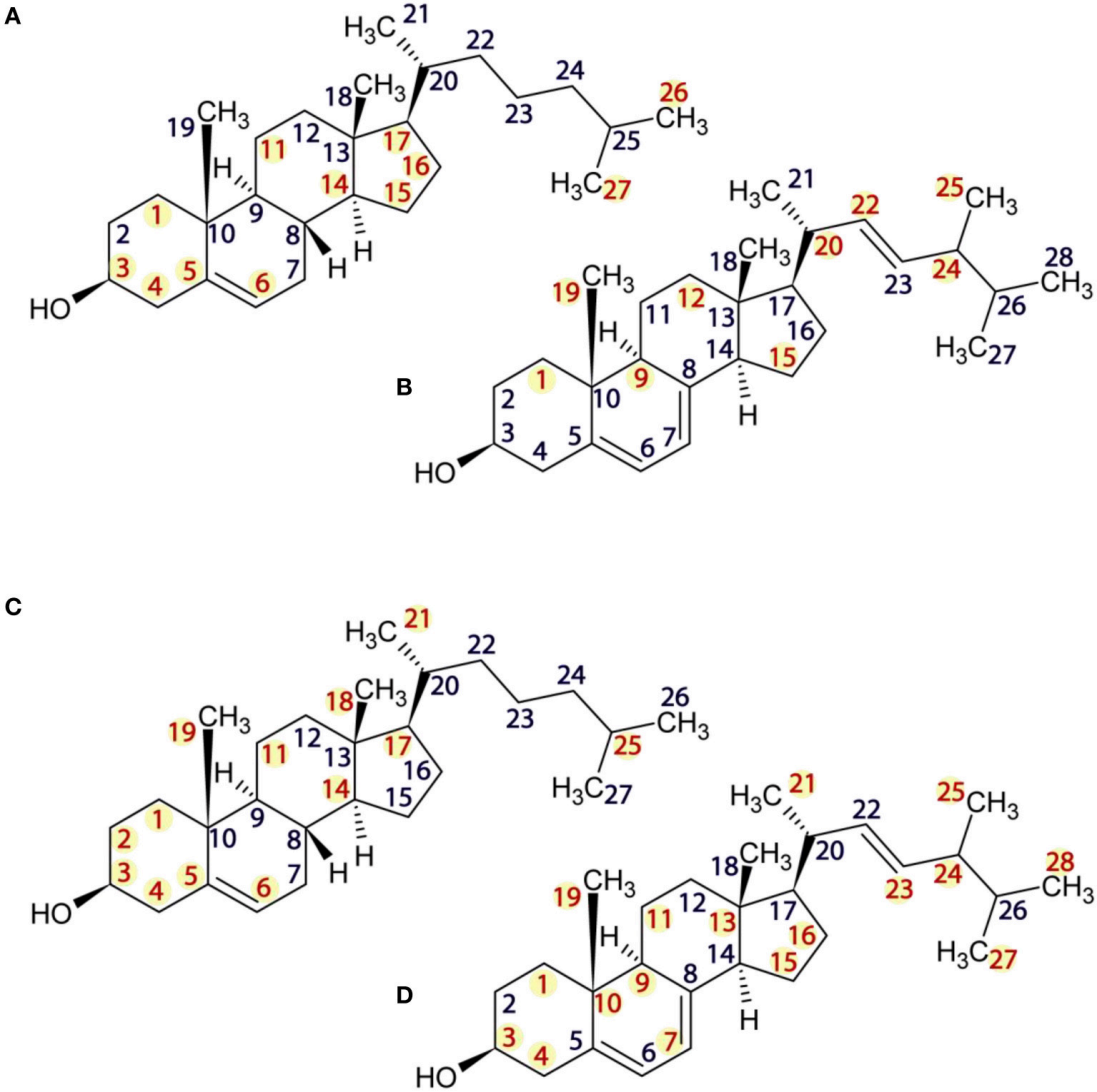
**Structures of cholesterol (A,C) and ergosterol (B,D) showing sites of ^**13**^C NMR spectral attenuation amphotericn B (A,B) and natamycin (C,D)**.

High resolution solid state ^13^C MAS NMR from natural abundance ^13^C is a useful tool for investigating drug-target interactions in lipid membranes at the molecular level. Here, we have examined the impact of amphotericin B or natamycin addition on mixed membranes of DOPC/cholesterol or ergosterol. Both polyene antimicrobials were observed to interact specifically with the sterol component and to impact less the phospholipid. Evidence of changes in sterol core and chain mobility indicates some degree of similarity between the molecular complex formation between amphotericin B or natamycin and either sterol. However, amphotericin B appears to engage cholesterol fairly flat on the ring structure without affecting ring methyls (Figure 7A) and to engage the chain end. By contrast, its interaction with ergosterol does highlight changes in ring methyl 19 and shows engagement of the chain double bond, again without affecting chain methyl 21 (Figure 7B). Natamycin interacts with both sterols engaging strongly ring A of cholesterol but less the remaining sterol ring, all ring methyls, which point to one side of the sterol core, as well as chain base methyl 21 and the end of the cholesterol chain (Figure 7C). Complex formation with ergosterol reveals a uniform sterol ring engagement with multiple contacts, comprehensive engagement of the sterol chain but not ring methyl 19 (Figure 7D). The most significant difference between the action of amphotericn B and natamycin is that the larger amphotericin B molecule engages lipid/sterol mixed membranes in a cooperative fashion, affecting the organization and stability of the membrane, while natamycin has a strong and specific interaction with both sterols. The comparatively low cooperativity of molecular interactions in natamycin-containing membranes allows unperturbed phospholipid/cholesterol membrane structure and functionality. Given the low therapeutic concentration of natamycin by comparison to membrane cholesterol, specific interactions affect little the available cholesterol fraction, which explains the low toxicity of natamycin. By contrast, lower ergosterol molarity is critically affected by natamycin causing deregulation of ergosterol-dependent protein function.

## Author contributions

FC-Prepared samples; Performed Experiments; Wrote the paper. DG-Prepared samples; Performed Experiments. JL-Prepared Samples; Performed Experiments; Wrote the paper. MR-Project Partner; Participated in Experimental Design. JD-Project Partner; Prepared and Supplied Samples. BB-Project lead; Designed the study; Wrote the paper.

### Conflict of interest statement

The authors declare that the research was conducted in the absence of any commercial or financial relationships that could be construed as a potential conflict of interest.
